# Carnosic Acid Induces Apoptosis and Inhibits Akt/mTOR Signaling in Human Gastric Cancer Cell Lines

**DOI:** 10.3390/ph14030230

**Published:** 2021-03-08

**Authors:** Waseem El-Huneidi, Khuloud Bajbouj, Jibran Sualeh Muhammad, Arya Vinod, Jasmin Shafarin, Ghalia Khoder, Mohamed A. Saleh, Jalal Taneera, Eman Abu-Gharbieh

**Affiliations:** 1 Department of Basic Medical Sciences, College of Medicine, University of Sharjah, Sharjah 27272, United Arab Emirates; welhuneidi@sharjah.ac.ae (W.E.-H.); kbajbouj@sharjah.ac.ae (K.B.); jmuhammad@sharjah.ac.ae (J.S.M.); jtaneera@sharjah.ac.ae (J.T.); 2 Sharjah Institute for Medical Research, University of Sharjah, Sharjah 27272, United Arab Emirates; arya.vinodnadat@gmail.com (A.V.); jsalam@sharjah.ac.ae (J.S.); 3 Department of Pharmaceutics and Pharmaceutical Technology, College of Pharmacy, University of Sharjah, Sharjah 27272, United Arab Emirates; gkhoder@sharjah.ac.ae; 4 Department of Clinical Sciences, College of Medicine, University of Sharjah, Sharjah 27272, United Arab Emirates; mohamed.saleh@sharjah.ac.ae; 5 Department of Pharmacology and Toxicology, Faculty of Pharmacy, Mansoura University, Mansoura 33516, Egypt

**Keywords:** carnosic acid, gastric cancer, apoptosis, Akt, mTOR, caspase, PARP, survivin

## Abstract

Gastric cancer is among the most common malignancies worldwide. Due to limited availability of therapeutic options, there is a constant need to find new therapies that could target advanced, recurrent, and metastatic gastric cancer. Carnosic acid is a naturally occurring polyphenolic abietane diterpene derived from *Rosmarinus officinalis* and reported to have numerous pharmacological effects. In this study, the cytotoxicity assay, Annexin V-FITC/PI, caspases 3, 8, and 9, cell cycle analysis, and Western blotting were used to assess the effect of carnosic acid on the growth and survival of human gastric cancer cell lines (AGS and MKN-45). Our findings showed that carnosic acid inhibited human gastric cancer cell proliferation and survival in a dose-dependent manner. Additionally, carnosic acid is found to inhibit the phosphorylation/activation of Akt and mTOR. Moreover, carnosic acid enhanced the cleavage of PARP and downregulated survivin expression, both being known markers of apoptosis. In conclusion, carnosic acid exhibits antitumor activity against human gastric cancer cells via modulating the Akt-mTOR signaling pathway that plays a crucial role in gastric cancer cell proliferation and survival.

## 1. Introduction

Worldwide, gastric cancer (GC) is considered to be one of the most common malignancies, leading to significant annual human cancer deaths [1]. GC is the third largest cause of cancer-related deaths and is a global public health priority [2]. While certain GC subtypes are diminishing due to improved screening, diagnosis, and healthcare services, other subtypes of GC are increasing in their incidence, such as non-cardiac GC in adults [3]. However, the prognosis for advanced, recurrent, and metastatic GC is still unsatisfactory and have very limited treatment options. Therefore, there is an urgent need to find novel and alternative therapies that could target gastric cancer.

**Figure 1:**
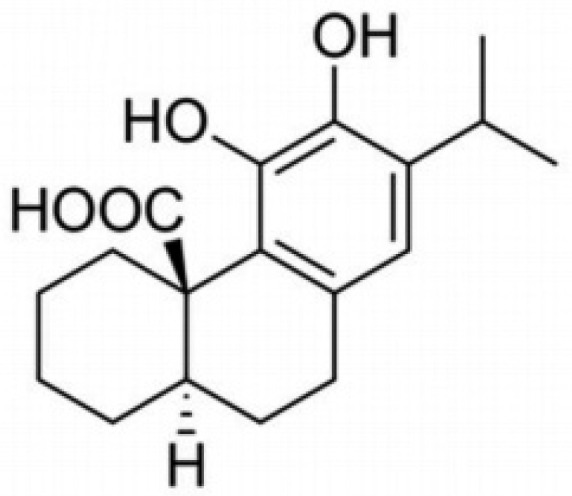
The chemical structure of carnosic acid.

Secondary metabolites derived from natural sources, primarily herbal plants, with potential pharmacological properties and less toxicity could be used to synthesize novel pharmaceutical products [4]. Among these natural compounds are the ‘terpenes’ that have been reported to possess various anticancer pharmacological properties. Diterpenes are a promising group of terpenes that are abundant in nature and are found in a wide variety of plant extracts and animal fats [4]. Carnosic acid is a naturally occurring polyphenolic abietane diterpene, as shown in Figure 1, commonly derived from herbal plants like *Rosmarinus officinalis*. The molecule’s pharmacological properties, including antitumor, antiviral, and anti-inflammatory activities, have been reported [5,6,7,8,9]. Previously it was reported that carnosic acid inhibited cell growth and induced cell cycle arrest in B16F10 melanoma cells in addition to the stimulation of p21 expression [5]. It has also been reported that carnosic acid inhibited the growth of estrogen receptor (ER)-negative human breast cancer cells by inducing G1-cell cycle arrest [10]. Carnosic acid showed strong anticancer activity in human cervical cancer cells by inhibiting cell growth and increased the production of reactive oxygen species (ROS) [11]. Furthermore, it downregulated the expression of cyclin A1 in both leukemia and colon cancer cells [12]. Additionally, carnosic acid was reported to induce apoptosis in various other cancer cell lines, including human prostate cancer, neuroblastoma, and hepatocellular carcinoma [9,13,14]. While in human hepatoma cells, it inhibited cell growth via targeting the Akt/mTOR pathway leading to autophagy induction [15].

The antitumor activity of carnosic acid on gastric cancer has never been previously investigated. Therefore, this study aimed to investigate the potential anti-proliferative and anticancer activity of carnosic acid on human gastric cancer cell lines, namely AGS and MKN-45, and explore the underlying molecular mechanism.

## 2. Results

### 2.1. Cytotoxic Activity

**Figure 2:**
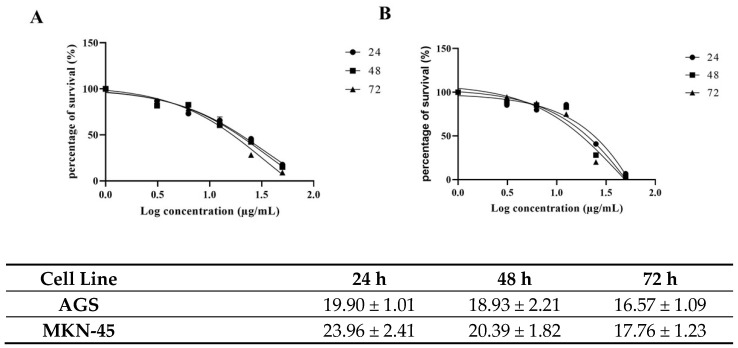
Effects of the carnosic acid on (**A**) AGS and (**B**) MKN-45 cells’ viability at three different incubation times (24, 48, and 72 h).

To investigate the effects of carnosic acid on gastric cancer cell proliferation, the AGS and MKN-45 cells were treated with increasing concentrations of carnosic acid (0, 1, 10, 25, 50, 100, and 200 µg/mL) for 24, 48, and 72 h. Carnosic acid treatment decreases cell viability in both cell lines in a dose-dependent manner (Figure 2). In AGS cells, the IC_50_ were 19.90, 18.93, and 16.57 µg/mL after 24, 48, and 72 h incubation, respectively, as shown in Figure 2A. In MKN-45 cells, carnosic acid has IC_50_ values of 23.96, 20.39, and 17.76 µg/mL after 24, 48, and 72 h incubation, respectively, as shown in Figure 2B. The concentrations of carnosic acid at 20 and 25 µg/mL for 24 h were selected for further analysis in AGS and MKN-45 cell lines, respectively, as they were the best representation of IC_50_.

### 2.2. Apoptotic Activity

Annexin V-FITC/PI double staining assay was used to assess carnosic acid’s proapoptotic activity on AGS and MKN-45 cells that were treated with 20 and 25 µg/mL of carnosic acid for 24 h, respectively.

**Figure 3:**
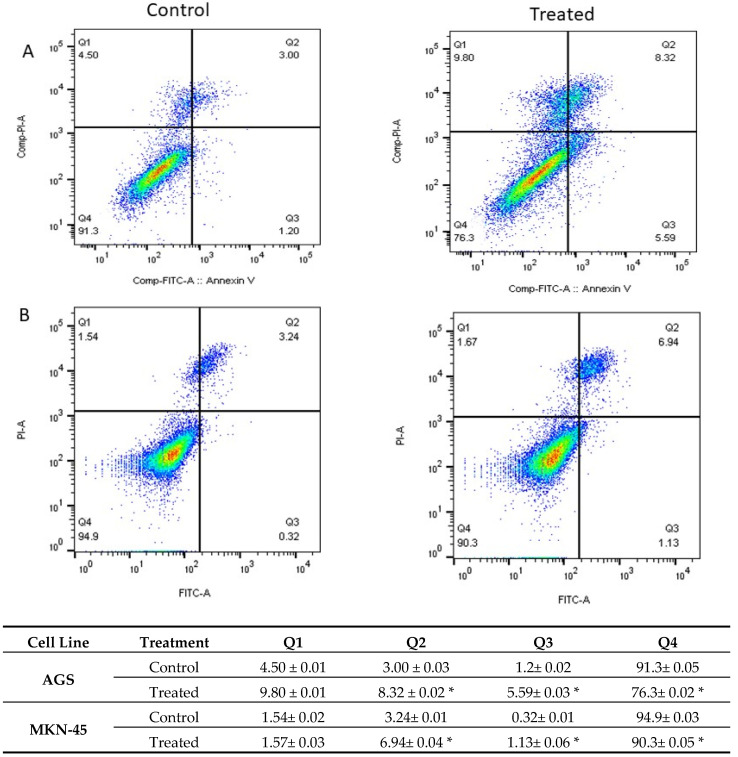
The level of apoptosis and necrosis was assessed by flow cytometry in (**A**) AGS and (**B**) MKN-45 cells, using Annexin V/PI staining. The control groups were without any treatment, and the treated group was treated with 20 and 25 μg/mL carnosic acid in AGS and MKN-45 cell lines, respectively for 24 h. Q1, Q2, Q3, and Q4 represent necrotic cells, late apoptotic, early apoptotic, and living cells, respectively. * *p* < 0.001.

The Annexin V-positive rate revealed that apoptotic cells increased significantly by treating the cells with carnosic acid (*p* > 0.001), as shown in Figure 3. The early and late apoptotic rates in AGS treated cells were 5.59 and 8.32, respectively, compared to 1.2 and 3.00, respectively, in the control.

### 2.3. Caspase Activity

**Figure 4:**
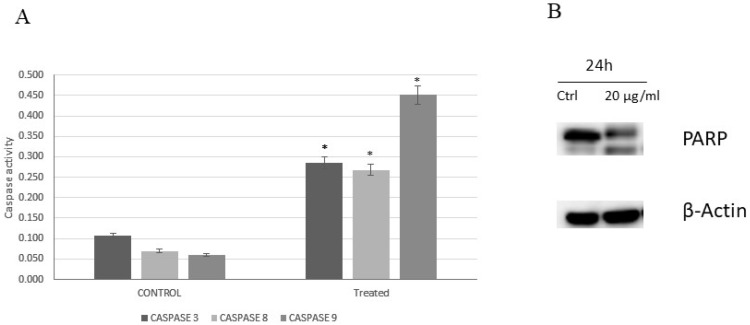
(**A**) Caspases 3, 8, and 9 activity on AGS cells. The control group received no treatment, and the treated group was treated with 20 µg/mL of carnosic acid for 24 h, * *p* < 0.001. (**B**) The effect of carnosic acid on the cleavage of PARP analyzed by Western blotting. The cells were treated with vehicle (0.1% DMSO) or treated with carnosic acid at 20 µg/mL for 24 h.

To assess carnosic acid’s apoptotic effect in AGS cells, the intracellular apoptotic molecular biochemical events were investigated. Figure 4A revealed that the activities of caspases 3, 8, and 9 were significantly stimulated in the cells treated with carnosic acid at 20 µg/mL for 24 h compared to untreated control cells. In addition, PARP cleavage was also noted in the treated cells using 20 µg/mL carnosic acid for 24 h, as shown in Figure 4B.

### 2.4. Cell Cycle Analysis

**Figure 5:**
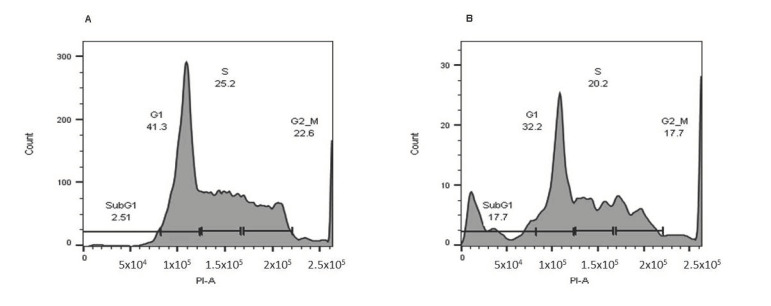
Carnosic acid induces cell cycle arrest in AGS cells. (**A**) AGS cells treated with DMSO (negative control) and (**B**) AGS cells treated with the carnosic acid (20 µg/mL) for 24 h. The cell-cycle analysis was performed using flow cytometry, and propidium was used to evaluate the DNA content.

To assess carnosic acid’s impact on cell cycle progression in AGS cells, the DNA content was measured by flow cytometry. Figure 5 shows that after 24 h of treatment with 20 µg/mL carnosic acid, there was a marked seven-fold increase in the sub-G1 population in AGS cells, which confirmed apoptosis induction after 24 h. Moreover, treatment with carnosic acid led to a noticeable reduction in both G1 and G2-M phase populations.

### 2.5. Western Blot

**Figure 6:**
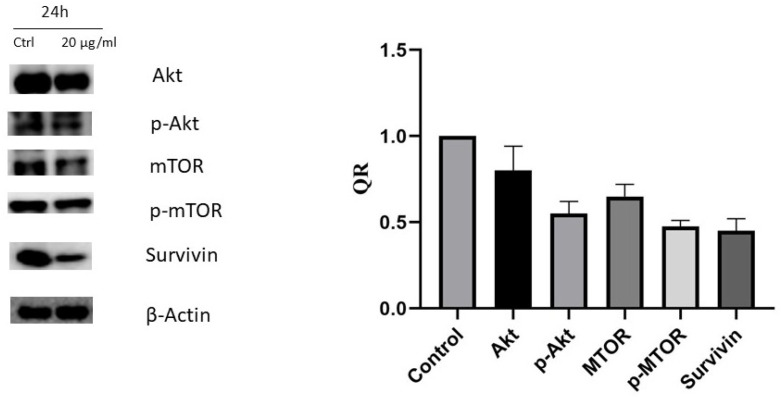
The effect of the carnosic acid on protein expression of AKT1, Phospho-AKT-S473, mTOR, Phospho-mTOR-S2448, and survivin analyzed by Western blotting. The cells were treated with vehicle (0.1% DMSO) or treated with carnosic acid at 20 µg/mL for 24 h. The quantitative analysis of each band density after normalization to the control is represented by QR.

To investigate the molecular mechanisms involved in apoptotic induction, the expression of several proteins regulating cell death—Phospho-mTOR-S2448, mTOR Phospho-AKT-S473, AKT1, and survivin—were assessed using Western blotting. The cells were treated with vehicle (0.1% DMSO) or with carnosic acid at 20 µg/mL for 24 h, representing the IC_50_ concentration. The findings confirmed a significant reduction in Phospho-mTOR-S2448, mTOR, Phospho-AKT-S473, AKT1, and survivin by 53, 33, 30, 27, and 57%, respectively, when treated with 20 µg/mL carnosic acid for 24 h, as shown in Figure 6.

## 3. Discussion

The anticancer activity of rosemary has been investigated in several types of cancer [16]. Carnosic acid, a major derivative of rosemary, has been linked with various biological activities, including antioxidant effects, epigenetic modification, anti-inflammatory activity, and regulation of immune systems [17].

To the best of our knowledge, this is the first study to demonstrate the growth inhibitory activity of carnosic acid on gastric cancer cells with IC_50_ values of 19.90 and 23.96 µg/mL after 24 h incubation on AGS and MKN-45 cells, respectively. It is worth mentioning that carnosic acid is reported not to affect normal human fibroblast cell viability [18].

To investigate the mechanism by which carnosic acid induces gastric cancer cell death, the Annexin V-FITC/PI assay was performed. Analysis of AGS and MKN-45 cells by flow cytometry showed that carnosic acid induced a shift in the cell population towards apoptosis, and the early and late apoptotic rates increased significantly relative to the control group (*p* < 0.001). It is noted that the cells responded differently to carnosic acid with respect to necrosis. The observed necrotic effect of carnosic acid on AGS cells can be attributed to the possible necroptotic effect, and such effect has been reported previously on other cell lines [19,20].

Apoptosis is regulated via several pathways, of which caspase induction is considered one of the main paths. Caspase activation analysis in this study was performed on caspases 3, 8, and 9 to elaborate both intrinsic and extrinsic pathways. The findings showed that carnosic acid activated all three caspases with the most remarkable activation being that of caspase 9. This suggests that the proapoptotic activity of carnosic acid is mediated through both extrinsic and intrinsic cell death pathways. The cross-communication between the intrinsic and extrinsic pathways is mediated by the cleavage of the proapoptotic Bid (Bcl-2 family member) and explains the marked activation of caspase 9 [21,22]. We also analyzed cleavage of the PARP protein, which is known for being cleaved by activated caspase 3 and, therefore, is essential to the apoptotic pathway [23]. Higher cleaved PARP protein levels (89 kDa) were detected in carnosic acid-treated cells, while only full-length PARP protein (116 kDa) was observed in the untreated cells.

Cell cycle analysis was performed to explore the molecular mechanisms underlining the observed cytotoxicity and proapoptotic activity of carnosic acid. The results revealed the ability of carnosic acid to induce G1-phase cell cycle arrest on AGS treated cells.

The obtained data are consistent with previously published reports that showed the anti-proliferative activity of carnosic acid on other cancer cell lines [5]. For example, carnosic acid was found to have an antitumor effect and inhibit the growth of estrogen receptor (ER)-negative human breast cancer cells and human cervical cancer cells in addition to prostate cancer, neuroblastoma, and hepatocellular carcinoma [10,11,13,14,24]. Moreover, carnosol, a phenolic diterpene isolated from rosemary and structurally related to carnosic acid, has been reported to suppress patient-derived gastric tumor growth by targeting RSK2 [25].

The PI3K/Akt/mTOR pathway is known to be commonly upregulated in various cancers, including gastric cancer [26,27]. Many studies have reported overactivation of the PI3K/AKT/mTOR pathway and high levels of phosphorylated/activated Akt in gastric cancer [24,25]. PI3K is a lipid kinase that phosphorylates membrane phospholipids upon its activation, leading to formation of 3-phosphoinositides, mainly phosphatidylinositol-3,4,5-triphosphate (PIP3). PIP3 plays a crucial role in the activation of Akt (the serine/threonine-protein kinase), which is an important promoter of the proliferation and survival of cells [28]. As a consequence of activated Akt, the mammalian target of rapamycin (mTOR) will be activated [29], which will increase protein synthesis and cell proliferation and will inhibit apoptosis [29,30,31]. Accordingly, the expression of the five proteins (AKT1, Phospho-AKT-S473, mTOR, Phospho-mTOR-S2448, and survivin) were studied based on their contribution to cell growth and proliferation.

Our results showed that carnosic acid significantly downregulated the expression of AKT1, Phospho-AKT-S473, mTOR, and Phospho-mTOR S2448 and survivin proteins, which explains the observed cell cycle arrest along with the proapoptotic activity of the acid on the tested cell line.

mTOR is a signaling molecule that promotes protein synthesis, cell survival, and proliferation, which is usually found activated in cancer [31,32]. Its activation in cancer cells is correlated with a high rate of protein synthesis and autophagy suppression [33,34]. Concurring with these findings, the obtained results confirmed the high levels of phosphorylation/activation of mTOR in untreated AGS cells. While treating the cells with carnosic acid exhibits very potent inhibition of mTOR expression and phosphorylation, this effect could be attributed to the observed inhibition of Akt, an mTOR activator, or through downregulation of other signaling components involved in the mTOR activation pathway [35]. Moreover, reduction in the phosphorylated/activated Akt and mTOR proteins’ expression may result in cancer cells being more sensitized to chemotherapies and reducing drug resistance [35]. This finding can be used as a base for future research into carnosic acid’s possible use with other chemotherapies used in gastric cancer to improve their efficacy.

Survivin is a protein that acts as a key regulator of cell proliferation and suppression of apoptosis. It is known for not being expressed in normal differentiated tissues but is highly upregulated in most tumors [36]. The overexpression of survivin is usually accompanied by angiogenesis, apoptosis inhibition, and cell proliferation activation [37]. Moreover, in the pre-mitotic phase, survivin binds to spindle microtubules leading tumor cells to escape G_2_/M phase monitoring [38]. This study’s findings prove that treatment with carnosic acid downregulates survivin and contributes to the anticancer effect of carnosic acid through activation of apoptosis and inhibition of cell proliferation.

## 4. Materials and Methods

### 4.1. Chemicals

The following chemicals were purchased from Sigma-Aldrich (St. Louis, MO, USA): MTT [3-(4,5-dimethylthiazol-2-yl)-2,5-diphenyltetrazolium bromide tetrazolium], ethanol, and dimethyl sulfoxide (DMSO). The ELISA kits for caspase assays, in addition to the Annexin V-FITC and PI staining assay, were purchased from Abcam (Cambridge, UK).

### 4.2. Cell Lines

Two human gastric adenocarcinoma cell lines AGS (CVCL 0139) and MKN-45 (DSMZ ACC409) were purchased from CLS (Cell Lines Service, Germany) and DSMZ (Deutsche Sammlung von Mikroorganismen und. Zellkulturen, Germany), respectively. The DMEM/RPMI medium supplemented with 10% heat-inactivated fetal bovine serum, 10,000 units/mL penicillin, and 10 mg/mL streptomycin was used to propagate the cells that were maintained at 37 °C and 5% CO_2_ atmosphere.

### 4.3. Cytotoxicity Assay

Carnosic acid was used at different concentrations ranging from 1 to 200 μg/mL. Cells were seeded in a 96-well plate (5 × 10^3^ cells/well) and incubated with carnosic acid for 24, 48, and 72 h. After treatment, MTT solution was added to the medium, and the cells were incubated at 37 °C for 2 h. The solubilization of MTT crystals was accomplished by adding 100 μL of DMSO followed by a 10 min incubation. Absorbance was measured using a microtiter plate reader at 570 nm. The rate of proliferation was calculated by comparing the absorption of treated cultures with untreated control cultures.

### 4.4. Annexin V/PI

The apoptosis induction was detected using the Annexin-V-PI staining method. In brief, the cells were incubated with 20 μg/mL carnosic acid for 24 h. Then, they were harvested, washed with PBS, and stained for 20 min using the Annexin V-FITC Kit. Finally, the flow cytometer (BD FACS Aria III; Becton Dickinson) was used to analyze the cells for apoptosis at 488 nm excitation, and a 530/30 nm bandpass filter was used for fluorescein detection. PI-positive cells were considered necrotic; cells positive for annexin V staining were considered early apoptotic, while cells positive for both annexin V and PI were considered late apoptotic. *FlowJo* software (Tree Star, Ashland, OR, USA) was used to analyze flow cytometry data.

### 4.5. Caspase 3, 8, and 9 Assays

Following the manufacturer’s protocol, briefly, AGS cells were incubated with 0.1% DMSO or carnosic acid at a concentration of 20 µg/mL for 24 h for control and treated cells, followed by mechanical disruption. Total protein was isolated and quantified by nanodrop. Fifty micrograms of total protein was used to estimate the activity of caspases 3, 8, and 9 at 400 nm absorbance. The percentage of activity was calculated by comparing the absorbance of the treated cells with the untreated ones.

### 4.6. Cell Cycle Analysis

The distribution of different phases of the cell cycle was analyzed using flow cytometry. In brief, the carnosic acid-treated AGS cells (20 μg/mL for 24 h) were harvested and fixed at −20 °C overnight using 1 mL of 70% ethanol. After washing with PBS, the cells were stained with PI and DNase-free RNase solution for 30 min. Cells and their progression through various cell cycle phases were then analyzed using a flow cytometry platform.

### 4.7. Western Blotting Analysis

An ice-cold NP40 lysis buffer containing protease cocktail-inhibitor (Sigma-Aldrich) was used to lyse both the carnosic acid-treated and untreated AGS cells. Thirty micrograms of protein was separated using 12% SDS-PAGE and blotted onto a nitrocellulose membrane (Bio-Rad, Hercules, CA, USA). Skimmed milk was used to block the membrane, followed by washing with tris-buffered saline with 0.1% tween solution (TBST). The membrane was incubated with primary IgG-unlabeled antibodies of AKT1 (#A17909), Phospho-AKT-S473 (#AP0637), mTOR (#A2445), and Phospho-mTOR-S2448 (#AP0115), purchased from ABclonal technology (Woburn, MA, USA), and survivin (EPR2675) (ab134170) purchased from Abcam, Cambridge, MA, USA, at 4 °C overnight. Later, the membrane was incubated with secondary antibodies (anti-mouse and anti-rabbit; Cell Signalling Technology) at 1:1000 dilutions for one hour. Chemiluminescence (Thermo Fisher Scientific, Waltham, MA, USA) and Image Lab software (ChemiDoc Touch Gel and Western blot imaging system; Bio-Rad) were used to detect the bands and quantify band-density, respectively. β-actin was used as a normalization control.

### 4.8. Statistical Analysis

Prism (GraphPad, V8, San Diego, CA, USA) was used for statistical analyses. The Comparisons between treated and untreated cells were conducted using student’s t-test, and a *p*-value of <0.05 was considered significant. The dose–response curves were used to calculate the IC_50_ values. All experiments were performed in triplicate, and data are expressed as the mean ± standard deviation (SD).

## 5. Conclusions

Carnosic acid inhibits proliferation and induces apoptosis in human gastric cancer cell lines. Mechanistic analysis revealed that carnosic acid induced the expression of caspases 3, 8, and 9, and triggered apoptosis by affecting the Akt/mTOR pathway. This study’s findings provide a rationale to initiate in vivo studies to evaluate the efficacy of carnosic acid on animal models as a complement to the currently used chemotherapies against gastric cancer.

## Data Availability

Data are contained within the article.

## Abbreviation

AGS	Human gastric adenocarcinoma cell line
AKT	Protein Kinase B
DMSO	dimethyl sulfoxide
FITC	Fluorescein isothiocyanate
GC	Gastric cancer
MKN-45	Human gastric adenocarcinoma cell lines
mTOR	Mechanistic Target of Rapamycin
MTT	3-(4,5-Dimethylthiazol-2-yl)-2,5-diphenyltetrazolium bromide tetrazolium
PARP	Poly (ADP-ribose) polymerase (PARP)
PI	Propidium iodide
ROS	reactive oxygen species
